# Dynamic walking features and improved walking performance in multiple sclerosis patients treated with fampridine (4-aminopyridine)

**DOI:** 10.1186/s12883-015-0431-0

**Published:** 2015-09-24

**Authors:** Philipp M. Keune, Adam J. Cocks, William R. Young, Janina M. Burschka, Sascha Hansen, Ulrich Hofstadt-van Oy, Patrick Oschmann, Jana Muenssinger

**Affiliations:** Department of Neurology, Klinikum Bayreuth GmbH, Hohe Warte 8, 95445 Bayreuth, Germany; Department of Physiological Psychology, Otto-Friedrich-University Bamberg, Bamberg, Germany; Department of Life Sciences, Brunel University London, London, UK; Department of Clinical Sciences, Brunel University London, London, UK; Department of Neurology, Klinikum Westfalen, Dortmund, Germany

**Keywords:** Multiple sclerosis (MS), Fampridine, 4-aminopyridine, Walking capacity, Walking dynamics, Linear deceleration, 6-minute walk, 25-foot-walk

## Abstract

**Background:**

Impaired walking capacity is a frequent confinement in Multiple Sclerosis (MS). Patients are affected by limitations in coordination, walking speed and the distance they may cover. Also abnormal dynamic walking patterns have been reported, involving continuous deceleration over time. Fampridine (4-aminopyridine), a potassium channel blocker, may improve walking in MS. The objective of the current study was to comprehensively examine dynamic walking characteristics and improved walking capacity in MS patients treated with fampridine.

**Methods:**

A sample of *N* = 35 MS patients (EDSS median: 4) underwent an electronic walking examination prior to (Time 1), and during treatment with fampridine (Time 2). Patients walked back and forth a distance of 25 ft for a maximum period of 6 min (6-minute 25-foot-walk). Besides the total distance covered, average speed on the 25-foot distance and on turns was determined separately for each test minute, at Time 1 and Time 2.

**Results:**

Prior to fampridine administration, 27/35 patients (77 %) were able to complete the entire 6 min of walking, while following the administration, 34/35 patients (97 %) managed to walk for 6 min. In this context, walking distance considerably increased and treatment was associated with faster walking and turning across all six test minutes (range of effect sizes: partial eta squared = .34-.72). Importantly, previously reported deceleration across test minutes was consistently observable at Time 1 and Time 2.

**Discussion:**

Fampridine administration is associated with improved walking speed and endurance. Regardless of a treatment effect of fampridine, the previously identified, abnormal dynamic walking feature, i.e. the linear decline in walking speed, may represent a robust feature.

**Conclusions:**

The dynamic walking feature might hence be considered as a candidate for a new outcome measure in clinical studies involving interventions other than symptomatic treatment, such as immune-modulating medication.

**Trial registration:**

DRKS00009228 (German Clinical Trials Register). Date obtained: 25.08.2015.

## Background

Multiple sclerosis (MS) is one of the most frequent progressive neurological diseases. It is often associated with impaired motor functioning due to an autoimmune response which corrupts myelinic sheaths of neurons of the central nervous system [1]. Among the resulting motor deficits, impaired walking ability represents a major confinement, interfering strongly with everyday life functioning [2–4].

### Treatment of walking disability with fampridine

Complementary to immune-modulating medication, symptomatic treatment may improve motor functioning. Fampridine (4-aminopyridine) has been shown to be an effective substance. It may prevent the release of potassium from potassium channels exposed due to the inflammatory demyelinating process [5]. Consequently, disrupted action potential conduction may be partly restored, yielding improvements in motor function and ambulation (for reviews see [6, 7]).

In their recent systematic review, Jensen et al. [6] report varying response rates to fampridine across studies. While some negative results with regards to a specific effect on ambulation and fatigue were reported [8, 9], analyses of subgroups of responders have predominantly confirmed positive findings, with response rates ranging between 35 and 43 % [10–14]. According to Jensen et al. [6], results across studies are supportive of fampridine yielding an increase in walking speed of approximately 25 %, i.e. a clinically relevant effect. Convergent evidence has recently been provided in a placebo-controlled randomized trial, results of which indicated that fampridine treatment yielded consistent improvements in various measures addressing mobility and balance throughout a period of 6 months [15].

### The assessment of dynamic walking characteristics in MS

The majority of walking tests implemented in studies outlined above addressed performance on relatively short distances, e.g. the 25-foot-walk [16]. Short tests may be suitable in clinical settings to address general walking ability, whereas longer tests may provide information about symptomatic correlates and underlying physiologic processes [17–19]. A test of longer duration commonly used to assess walking disability in MS is the 6-minute walk, in which walking speed is monitored for 6 min [18, 20–22]. This test may also reveal impaired dynamic walking features. Burschka et al. [3] have identified an atypical velocity profile characterized by continuous deceleration throughout the test, relative to healthy controls. MS patients might hence be characterized by impaired walking dynamics involving a consistent linear decline in walking speed on the 6-minute walk.

### Purpose of the current study

Since improvements in ambulation related to fampridine treatment have predominantly been addressed by relatively short walking tests, the concurrent body of literature may be extended by the implementation of tests of longer duration. Further, the linear deceleration profile remains to be replicated and it remains to be examined whether the linear trend shows sufficient temporal stability. To date, sensitivity of the linear trend to alterations in other clinical parameters remains speculative. Yet, a replication and an exploration of its properties might provide initial information on whether it might be considered as a candidate parameter in intervention studies. In this context, it appears sensible to examine, whether the linear trend is consistently observable in patients treated with fampridine.

In the current study, MS patients completed an automated 6-minute walking test which required them to walk back and forth a distance of 25 ft. It was assumed that (a) prior to administration of fampridine, MS patients would display overall lower walking speed and cover a shorter total distance than during treatment with fampridine. Moreover, it was assumed that (b) the previously reported linear decline in speed throughout the test could be consistently replicated at both assessment points. The latter assumption was derived from findings reported by Burschka et al. [3], who observed the atypical linear trend in both, moderately (Expanded Disability Status Scale, EDSS >3.5) and mildly disabled patients (EDSS <4). Subsequently, it was examined whether deceleration trends differed across assessment points, in an exploratory analysis.

## Methods

### Study design and participants

The current study was approved by the ethical committee of the University of Bayreuth, Germany. All participants provided written informed consent. A sample of 35 adult MS patients was recruited in the Deparment of Neurology, Klinikum Bayreuth GmbH. Patients who were about to start treatment, or who already received fampridine, were contacted by a study nurse. Patients were eligible to participate in the study in case of a confirmed MS diagnosis [23], indication for fampridine treatment, and the ability to continuously walk for at least 3 min according to self-report. Patients scheduled for fampridine treatment were tested prior to administration of a daily dose of twice 10 mg of Fampyra® (Time 1) as well as within 1 week following the initial test (Time 2). Patients who already received fampridine could also participate if they reported to be interested in detailed information about a potential effect of their treatment. In this case, patients could pause their daily dose of twice 10 mg of Fampyra® and, after a washout period of at least 24 h, be tested off and on fampridine at Time 1 and Time 2. Sample characteristics, clinical information and demographics are displayed in Table 1.

**Table 1:** Sample description (*N* = 35)

Demographics	
Age M (SD)	54.34 (9.85)
Gender	27 female, 8 male
Clinical information	
MS type (N)	
Relapsing remitting (RR-MS)	3 (9 %)
Secondary progressive (SP-MS)	26 (74 %)
Primary progressive	6 (17 %)
EDSS score, Mdn (range)	4 (4–7)
Illness duration in years, M (SD)	14.56 (7.95)
Health behavior	
Smoking (yes/no)	10/25
Physical activity per week (N)	None: 10
	1–3 times: 17
	4 times: 5
	>4 times: 2

Physical activity was defined as any sport/exercise-related activity patients engaged in on a regular basis, at least once per week

*Mdn* Median, *M* Mean, *SD* Standard deviation

### Assessment of walking performance and data analysis

**Fig. 1:**
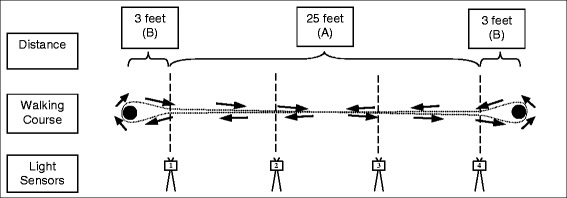
Illustration of the implemented walking test involving light sensors (1–4) and the examined straight distance (*A*) and curves (*B*). See methods section for details on the derivation of respective walking parameters

At each of the two assessment points, patients completed a walking test, which required them to repeatedly cover a distance of 25 ft throughout a maximal assessment period of 6 min. Patients were instructed to walk as enduring and fast as possible in context of their walking disability. An illustration of the general outline of the walking test is displayed in Fig. 1.

At each end of the 25-foot distance (A), a pole was placed, 3 ft away from the respective endpoint of the 25-foot distance (B). Four light sensors were placed parallel to the 25-foot distance, with which the exact timing of a patient passing the light sensor was recorded. As walking speed was continuously monitored throughout the walking test, the setup yielded the possibility to determine the average duration on the 25-foot walk (A) and the average duration for turns at the end of the 25-foot distance (B). The latter was defined as the time that passed between leaving and reentering the 25-foot distance at light sensors 1 and 4, respectively.

In an initial descriptive analysis, it was recorded, how many minutes of the maximum time of 6 min patients were able to walk at Time 1 and Time 2. Based on the previous suggestion, that an improvement in walking performance of approximately 25 % is of high clinical relevance [6], the number of patients whose total distance on the 6-minute walk increased by at least 25 %, or 20 % (two thresholds), was determined.

For the statistical analysis addressing the hypotheses outlined in the introduction, several parameters were derived for each participant at Time 1 and Time 2:

- *Total distance.* Firstly, as a global parameter, the total distance covered throughout the test was determined, including distances (A) and (B).
- 25-foot-walk_speed_. Secondly, the average time required to cover the 25-foot distance (A) was determined separately for each test minute. The derivation of this speed parameter occurred separately for each test minute, since this yielded the possibility to determine whether patients showed deceleration on the 25-foot distance across test minutes. For comparison see Burschka et al. [3].
- *Curve*_*speed*_. Finally, the average time to walk around the poles at the end of the 25-foot distance (B) was extracted as a speed indicator involving the coordinative quality required to make turns. As was the case for the 25-foot-walk_speed_ parameter, mean speed values were calculated separately for each test minute to evaluate putative deceleration throughout the walking test.

### Statistical analysis

Referring to hypothesis (a), a repeated measures analysis of variance (ANOVA) involving the within-subjects factor Time (Time 1 vs. Time 2) was utilized to evaluate a putative increase in the total distance covered between respective assessment points, i.e. off vs. on fampridine. Alterations in 25-foot-walk_speed_ and Curve_speed_ were evaluated with the same ANOVA model.

Additionally, the percentage of patients who were able to walk throughout the entire 6 min at Time 1 and Time 2 was assessed. As this analysis revealed that all patients managed to walk at least 4 min at Time 1, the subsequent analysis referring to hypothesis (b), was conducted separately for the first 4 min of the walking test, and for the entire 6 min of the test. To this end, in case of the 25-foot-walk_speed_ parameter, the statistical model included two within-subjects factors, i.e. Time (Time 1 vs. Time 2) and Minute (1–4/1–6). The same factors were included for the analysis of the Curve_speed_ parameter.

Based on hypothesis (a), a main effect of Time was expected, reflecting an increase in distance and speed (25-foot-walk_speed_, Curve_speed_) from Time 1 to Time 2. Following hypothesis (b), a significant main effect of Minute with a linear deceleration trend at Time 1 and Time 2 was assumed, in line with results of Burschka et al. [3]. Finally, referring to the exploratory analysis, it was examined whether a linear trend interaction Time × Minute occurred, indicative of a potentially steeper deceleration slope at Time 1 than at Time 2.

## Results

Prior to administration of fampridine (Time 1), 27/35 patients (77 %) were able to complete the entire 6 min of walking, while following the administration (Time 2), 34/35 patients (97 %) managed to walk for 6 min. In this context, 12/34 patients (35 %) showed an increase of at least 20 % in the total distance covered throughout the six test minutes, and 8/34 (24 %) showed an increase of at least 25 %. All 35 patients managed to walk for at least 4 min at Time 1 and Time 2.

### Quantitative analysis: total distance and average speed at Time 1 and Time 2

The total distance patients covered during the first 4 min of the walking test (*N* = 35, Table 2a) and during the entire 6 min (*N* = 27, Table 2b) significantly increased following fampridine administration. As displayed in the respective tables, this increase was characterized by highly significant main effects of Time, involving large effect sizes. For average 25-foot-walk_speed_ and Curve_speed_, similar effects were observed, involving consistent improvements.

**Table 2:** Alterations in walking performance between Time 1 and Time 2

(a)	Minute 1–4 (*N* = 35)			Statistic		
	Time 1	Time 2	% improvement	F	*p*	partial eta squared
Total distance (feet)						
Mean	758.5	935.2	23 %	49.46	<.001	.59
SD	274.7	253.7				
25-foot-walk_speed_						
Mean	10.21	8.41	18 %	21.25	<.001	.39
SE	0.77	0.52				
Curve_speed_						
Mean	5.55	4.50	19 %	17.34	<.001	.34
SE	0.44	0.30				
(b)	Minute 1–6 (*N* = 27)			Statistic		
	Time 1	Time 2	% improvement	F	*p*	Partial eta squared
Total distance (feet)						
Mean	869.0	993.6	14 %	65.82	<.001	.72
SD	200.3	226.0				
25-foot-walk_speed_						
Mean	9.06	7.99	12 %	59.36	<.001	.70
SE	0.60	0.58				
Curve_speed_						
Mean	4.95	4.31	13 %	77.91	<.001	.75
SE	0.35	0.35				

Comparison between walking performance at Time 1 (off fampridine) and Time 2 (on fampridine), displayed for test minutes 1–4 (a) and test minutes 1–6 (b). Mean speed values reflect the duration (in seconds) necessary to cover the 25-foot distance (25-foot-walk_speed_) and to circle the poles at the end of the 25-foot walk distance (Curve_speed_), averaged across test minutes 1–4 and 1–6, respectively. Note that the analysis occurred separately for the respective test minutes, due to the fact that all participants managed to complete the first 4 min of the test at both assessment points (*N* = 35), whereas data of 27 patients was available for the entire 6 min at Time 1

### Dynamic walking features within walking tests at Time 1 and Time 2

Walking and turning speed significantly decreased across test minutes. In case of the first four test minutes, this was reflected by a significant main effect of Minute on 25-foot-walk_speed_ [F(3,102) = 13.02, *p* <.001, partial eta squared = .28] and on Curve_speed_ [F(3,102) = 4.85, *p* = .003, partial eta squared = .125].

**Fig. 2:**
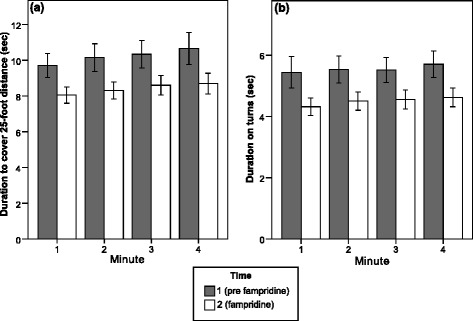
Improvements in walking speed (a) and curve speed (b) associated with treatment onset of fampridine during test minute 1–4 (N = 35). The linear deceleration trend, consistently observable at Time 1 and Time 2, was most salient on the 25-foot distance (a; see Table 3 for statistics). Error bars represent standard errors

Both main effects involved significant linear trends [25-foot-walk_speed_: F(1,34) = 14.82, *p* <.001, partial eta squared = .30; Curve_speed_: F(1,34) = 5.96, *p* = .02, partial eta squared = .15]. As displayed in Table 3 and Fig. 2, for minutes 1–4, significant linear deceleration trends on the 25-foot-walk_speed_ parameter emerged at Time 1 and Time 2. In case of the Curve_speed_ parameter, the linear deceleration trend did not reach significance at Time 1, whereas it was significant at Time 2 (Table 3).

**Table 3:** Linear deceleration trends during minutes 1–4

		Minute				Linear trend statistic		
		Min 1	Min 2	Min 3	Min 4	F	*p*	Partial eta squared
	25-foot-walk_speed_							
Time 1	Mean	9.70	10.15	10.33	10.66	12.80	.001	.27
	SD	4.02	4.57	4.53	5.25			
Time 2	Mean	8.05	8.30	8.60	8.69	12.48	.001	.27
	SD	4.02	4.57	4.53	5.25			
	Curve_speed_							
Time 1	Mean	5.44	5.54	5.51	5.71	2.11	.16	.06
	SD	3.06	2.58	2.43	2.56			
Time 2	Mean	4.32	4.50	4.56	4.63	10.08	.003	.23
	SD	1.70	1.76	1.81	1.82			

Linear deceleration on the speed parameters across minutes 1–4 at Time 1 and Time 2. Mean speed values reflect the average duration (in seconds) necessary to cover the 25-foot distance (25-foot-walk_speed_) and to circle the poles at the end of the 25-foot walk distance (Curve_speed_), during respective test minutes 1–4. With the exception of the Curve_speed_ parameter at Time 1, patients showed consistent deceleration characterized by significant linear trends. *SD* Standard deviation

When all six test minutes were analyzed at Time 1 and Time 2, a similar pattern of deceleration within respective tests emerged, involving significant main effects of Minute on 25-foot-walk_speed_ [F(5,130) = 12.80, *p* <.001, partial eta squared = .33] and on Curve_speed_ [F(5,130) = 7.28, *p* <.001, partial eta squared = .22]. In both cases, significant linear trend components were involved [25-foot-walk_speed_: F(1,26) = 18.68, *p* <.001, partial eta squared = .42; Curve_speed_: F(1,26) = 5.22, *p* = .001, partial eta squared = .17], which were observable at Time 1 and Time 2 (Table 4).

**Table 4:** Linear deceleration trends during minutes 1–6

		Minute						Linear trend statistic		
		Min 1	Min 2	Min 3	Min 4	Min 5	Min 6	F	*p*	Partial eta squared
	25-foot-walk_speed_									
Time 1	Mean	8.70	8.88	9.02	9.19	9.37	9.19	12.66	.001	.42
	SD	3.16	3.02	3.04	3.31	3.41	2.93			
Time 2	Mean	7.65	7.84	8.05	8.09	8.19	8.10	12.43	.002	.32
	SD	2.71	2.71	3.19	3.21	3.27	3.16			
	Curve_speed_									
Time 1	Mean	4.75	4.90	4.92	5.04	5.10	4.96	5.22	.031	.17
	SD	1.88	1.76	1.86	1.86	1.90	1.80			
Time 2	Mean	4.11	4.23	4.30	4.35	4.39	4.49	8.82	.006	.25
	SD	1.67	1.63	1.77	1.77	1.87	2.30			

Linear deceleration on the speed parameters across minutes 1–6 at Time 1 and Time 2. Mean speed values reflect the average duration (in seconds) necessary to cover the 25-foot distance (25-foot-walk_speed_) and to circle the poles at the end of the 25-foot walk distance (Curve_speed_), during respective test minutes 1–6. *SD* Standard deviation

### Exploratory analysis: deceleration trend at Time 1 and Time 2

The exploratory analysis addressing a comparison between the deceleration trend at Time 1 and Time 2 revealed that the Time × Minute interaction did not reach significance, neither in case of the model involving the first four test minutes, nor involving the entire six test minutes (all *p*-values >.05).

## Discussion

Impaired ambulation is a frequent phenomenon in MS patients. Besides limitations in walking speed and the total distance which may be covered, also dynamic walking features, such as continuous deceleration throughout longer walking tests have been examined [3]. Fampridine may exert beneficial effects on walking disability. Nevertheless, the concurrent body of literature may be extended by examining alterations in walking capacity related to fampridine on longer walking tests, and with regards to the atypical linear decline in walking speed.

### Improvements in walking capacity and speed

In the current study, fampridine treatment was associated with improvements in various walking parameters, including total distance covered and different speed parameters. Compatible with results of previous work, fampridine treatment appeared to involve a salient response, yielding an increase from 77 % of patients who were capable of finishing the 6-minute-walking test before treatment, in comparison to 97 % who were able to walk for 6 min during treatment. It is noteworthy that the involved increase in the total distance that patients were able to cover (23 %, Table 2a) during the first four test minutes, is congruent with recent estimates, according to which fampridine may improve walking capacity by 25 % [6]. Moreover, the latter clinical threshold was surpassed by 24 % of the patients of the current study on the 6-minute walking test. When a somewhat lower threshold of 20 % improvement in total distance covered was applied, 35 % of the sample surpassed that threshold. Hence, within the boundaries of methodological restrictions of the current work, a considerable number of putative responders could be identified. These findings may be regarded as generally supportive of the treatment and assessment methodology applied in the current study.

Nevertheless, as outlined in the limitations section in detail, it cannot be ruled out that walking improvements may have been confounded by a training effect. It also needs to be considered that in the quantitative analysis of data concerning the total distance covered, alterations were lower (14 %, Table 2b), when performance on all six test minutes was analyzed. In sum, these findings also imply considerable heterogeneity of alterations in walking performance across patients and modes of analysis. All further results of speed parameters hence need to be regarded cautiously, within the boundaries of these limitations.

The latter reasoning should also be followed when interpreting the results on walking speed and during turning (Curve_speed_). Highly significant improvements in coordinative ability required during walking of curves associated with fampridine treatment might be interpreted in favor of fampridine treatment. Nevertheless, potentially confounding factors, such as heterogeneity of responsiveness to fampridine within the sample, as well as a training effect, need to be taken into consideration.

### Dynamic walking features

As Burschka et al. [3] have noted, performance on the 6-minute-walk may reveal information about atypical dynamic walking features in MS. In the current study, linear deceleration trends were observed in case of 25-foot-walk_speed_ and Curve_speed_, at both assessment points, when patients were off and on fampridine. With the only exception of performance during the first four test minutes at Time 1, the linear deceleration trend was consistently observed. Based on findings reported by Burschka et al. [3], according to which deceleration in MS is atypical, relative to performance of healthy individuals, this implies that the linear trend might represent a candidate for a new marker of walking disability. Importantly, sensitivity of the linear trend to alterations in other clinical parameters remains speculative. However, in context of this basic methodological study, it is noteworthy that the linear trend appeared particularly robust in case of straight distances (25-foot-walk_speed_). It may also be detected for walking characteristics involving coordinative qualities (Curve_speed_). To our knowledge, the current work represents the first replication of findings reported by Burschka et al. [3]. Keeping the above-mentioned, general limitations of the current study in mind, the current results allow the suggestion that the linear deceleration trend may be explored as a candidate clinical marker in MS in future studies.

### Limitations

While the current findings are compatible with a putative treatment effect of fampridine and are promising with regards to the feasibility of the linear deceleration trend as a candidate for an outcome measure in future clinical studies, results need to be interpreted in the context of several limitations. Firstly, it needs to be pointed out that the current design did not involve a control group. Conceptually, without such a group, beneficial alterations in walking behavior, as observed in the current study, cannot be attributed to onset of fampridine treatment directly. Given the extensive body of literature in strong support of such an effect of fampridine [5], and the methodological emphasis of the current work, this limitation might not be critical. Nevertheless, the related, potentially confounding factor of a training effect on the walking test, as well as heterogeneity of assumed responsiveness to fampridine treatment and a small sample size make a careful interpretation of the results necessary.

## Conclusions

Within the boundaries of these limitations, the current study provides new information on the usefulness of an automated 6-minute 25-foot walking test for the detection of beneficial alterations in walking capacity and speed associated with fampridine treatment. Further, it provides the first replication of previous findings referring to the detection of the linear deceleration trend in MS patients in context of an intervention study. Based on the latter, the linear deceleration trend may be suggested as a candidate for a marker in the evaluation of walking performance in future studies.

## Abbreviations

M: Mean
Mdn: Median
MS: Multiple sclerosis
EDSS: Expanded disability status scale
SD: Standard deviation

## References

[1] Compston A, Coles A (2008). Multiple sclerosis. Lancet.

[2] Motl RW, Pilutti LA (2012). The benefits of exercise training in multiple sclerosis. Nat Rev Neurol.

[3] Burschka JM, Keune PM, Menge U, Hofstadt-van Oy U, Oschmann P, Hoos O (2012). An exploration of impaired walking dynamics and fatigue in multiple sclerosis. BMC Neurol.

[4] Burschka JM, Keune PM, Oy UH, Oschmann P, Kuhn P (2014). Mindfulness-based interventions in multiple sclerosis: beneficial effects of Tai Chi on balance, coordination, fatigue and depression. BMC Neurol.

[5] Krishnan AV, Kiernan MC (2013). Sustained-release fampridine and the role of ion channel dysfunction in multiple sclerosis. Mult Scler.

[6] Jensen HB, Ravnborg M, Dalgas U, Stenager E (2014). 4-Aminopyridine for symptomatic treatment of multiple sclerosis: a systematic review. Ther Adv Neurol Disord.

[7] Blight AR, Henney HR, Cohen R (2014). Development of dalfampridine, a novel pharmacologic approach for treating walking impairment in multiple sclerosis. Ann N Y Acad Sci.

[8] Bever CT, Young D, Anderson PA, Krumholz A, Conway K, Leslie J, Eddington N, Plaisance KI, Panitch HS, Dhib-Jalbut S (1994). The effects of 4-aminopyridine in multiple sclerosis patients: results of a randomized, placebo-controlled, double-blind, concentration-controlled, crossover trial. Neurology.

[9] Rossini PM, Pasqualetti P, Pozzilli C, Grasso MG, Millefiorini E, Graceffa A, Carlesimo GA, Zibellini G, Caltagirone C (2001). Fatigue in progressive multiple sclerosis: results of a randomized, double-blind, placebo-controlled, crossover trial of oral 4-aminopyridine. Mult Scler.

[10] Schwid SR, Petrie MD, McDermott MP, Tierney DS, Mason DH, Goodman AD (1997). Quantitative assessment of sustained-release 4-aminopyridine for symptomatic treatment of multiple sclerosis. Neurology.

[11] Goodman AD, Cohen JA, Cross A, Vollmer T, Rizzo M, Cohen R, Marinucci L, Blight AR (2007). Fampridine-SR in multiple sclerosis: a randomized, double-blind, placebo-controlled, dose-ranging study. Mult Scler.

[12] Goodman AD, Brown TR, Cohen JA, Krupp LB, Schapiro R, Schwid SR, Cohen R, Marinucci LN, Blight AR, Fampridine MS-F202 Study Group (2008). Dose comparison trial of sustained-release fampridine in multiple sclerosis. Neurology.

[13] Goodman AD, Brown TR, Krupp LB, Schapiro RT, Schwid SR, Cohen R, Marinucci LN, Blight AR, Fampridine MS-F203 Investigators (2009). Sustained-release oral fampridine in multiple sclerosis: a randomised, double-blind, controlled trial. Lancet.

[14] Goodman AD, Brown TR, Edwards KR, Krupp LB, Schapiro RT, Cohen R, Marinucci LN, Blight AR, MSF204 Investigators (2010). A phase 3 trial of extended release oral dalfampridine in multiple sclerosis. Ann Neurol.

[15] Hupperts R, Lycke J, Short C, Gasperini C, McNeill M, Medori R, et al. Prolonged-release fampridine and walking and balance in MS: randomised controlled MOBILE trial. Mult Scler. 2015. 10.1177/1352458515581436 PMC4749757 25921050

[16] Fischer JS, Jak AJ, Kniker JE, Rudick RA, Cutter G (2001). Multiple Sclerosis Functional Composite (MSFC): administration and scoring manual.

[17] Kieseier BC, Pozzilli C (2012). Assessing walking disability in multiple sclerosis. Mult Scler.

[18] Motl RW, Balantrapu S, Pilutti L, Dlugonski D, Suh Y, Sandroff BM, Lane A, Fernhall B (2013). Symptomatic correlates of six-minute walk performance in persons with multiple sclerosis. Eur J Phys Rehabil Med.

[19] Motl RW, Suh Y, Balantrapu S, Sandroff BM, Sosnoff JJ, Pula J, Goldman MD, Fernhall B (2012). Evidence for the different physiological significance of the 6- and 2-minute walk tests in multiple sclerosis. BMC Neurol.

[20] Goldman MD, Marrie RA, Cohen JA (2008). Evaluation of the six-minute walk in multiple sclerosis subjects and healthy controls. Mult Scler.

[21] Savci S, Inal-Ince D, Arikan H, Guclu-Gunduz A, Cetisli-Korkmaz N, Armutlu K, Karabudak R (2005). Six-minute walk distance as a measure of functional exercise capacity in multiple sclerosis. Disabil Rehabil.

[22] Chetta A, Rampello A, Marangio E, Merlini S, Dazzi F, Aiello M, Ferraro F, Foresi A, Franceschini M, Olivieri D (2004). Cardiorespiratory response to walk in multiple sclerosis patients. Respir Med.

[23] McDonald WI, Compston A, Edan G, Goodkin D, Hartung HP, Lublin FD, McFarland HF, Paty DW, Polman CH, Reingold SC, Sandberg-Wollheim M, Sibley W, Thompson A, van den Noort S, Weinshenker BY, Wolinsky JS (2001). Recommended diagnostic criteria for multiple sclerosis: guidelines from the International Panel on the diagnosis of multiple sclerosis. Ann Neurol.

